# Acute variceal bleeding in a patient with idiopathic myelofibrosis successfully treated with endoscopic variceal band ligation and chemotherapy: a case report

**DOI:** 10.1186/1752-1947-4-25

**Published:** 2010-01-28

**Authors:** Kumiko Tamaki, Michiro Otaka, Naoto Sakamoto, Kenshi Matsumoto, Shunhei Yamashina, Sumio Watanabe

**Affiliations:** 1 Department of Gastroenterology, Juntendo University School of Medicine, 2-1-1 Hongo, Bunkyo-Ku, Tokyo, 113-8421, Japan

## Abstract

**Introduction:**

Idiopathic myelofibrosis is a chronic myeloproliferative disorder characterized by leukoerythroblastosis, massive splenomegaly, and increases in the reticular and collagen fibers in the bone marrow. Portal hypertension is observed in some patients with idiopathic myelofibrosis. Gastrointestinal hemorrhages, which are due mostly to the rupture of the esophageal varices, have been sporadically reported to be an infrequent complication of idiopathic myelofibrosis.

**Case presentation:**

We report a case of a Japanese 63-year-old woman with myelofibrosis and variceal hemorrhage, with a background of concomitant portal and pulmonary hypertension. She was successfully treated through a combination of endoscopic variceal ligation and chemotherapy.

**Conclusion:**

This is the first known report on the successful application of endoscopic variceal ligation and chemotherapy as the therapeutic procedure for an esophageal variceal hemorrhage in a patient with myelofibrosis.

## Introduction

Myelofibrosis, one of the chronic myeloproliferative disorders, is characterized by a massive splenomegaly, leukoerythroblastosis, and increased reticular and collagen fibers in the bone marrow [1,2]. Portal hypertension has been observed in 10% to 17% of patients with idiopathic myelofibrosis (IMF) [1,2]. A gastrointestinal hemorrhage, which is due mostly to the rupture of the esophageal varices, has been sporadically reported to be an infrequent complication of IMF [3-6]. However, the management of variceal bleeding can be complicated as esophageal varices could be caused by portal hypertension, which is a complication of extramedullary hematopoiesis in IMF. In this case report, we present the usefulness of endoscopic variceal ligation and combined chemotherapy not only for variceal bleeding but also for the prevention of recurrence of esophageal varices.

## Case presentation

A 63-year-old Japanese woman was diagnosed with IMF in 1994 and was since treated with hydroxycarbamide. She was admitted to our hospital on January 22, 2008, with melena. On admission, her consciousness was clear. Her physical status was moderate and her nutrition was good. She had a blood pressure of 104/56 mmHg and a heart rate of 74 beats/min. Anemia was observed on her palpebral conjunctivae, and jaundice was observed on her bulbar conjunctivae. The laboratory findings were as follows: hemoglobin 7.5 g/dl, platelets 144 × 10^9^/L, white blood cells 2.52/μl, total protein 7.0 g/dl, and albumin 3.9 g/dl. Her peripheral blood smear pattern showed a possible presence of myeloproliferative disease such as immature myeloid cells, teardrop-shaped cells, and giant platelets.

**Figure 1:**
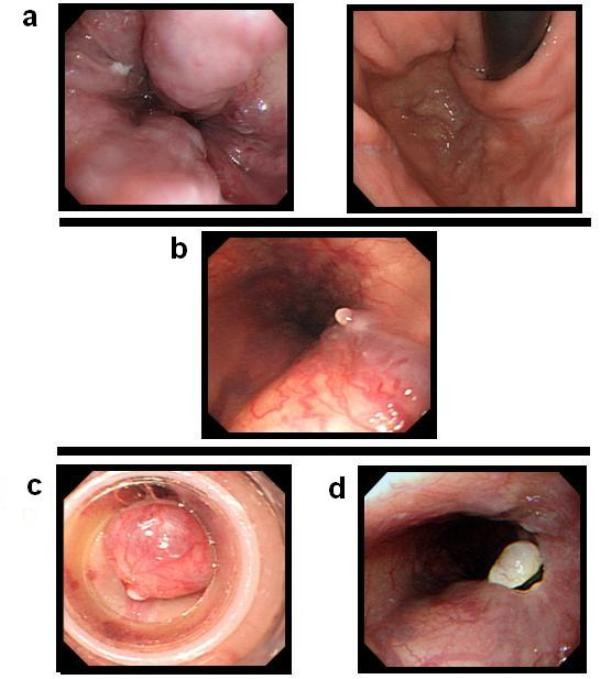
**Esophagogastroscopy on admission**. **(a) **Endoscopic picture shows esophagogastric varices (Li F2 Cb RC (+), Lg-cf F1 RC (-). **(b) **White thrombus was observed on the middle of the esophageal varices. **(c) **The first session of variceal ligation with one rubber band was carried out successfully. **(d) **Endoscopic picture shows the banded varices 5 days after the first variceal ligation.

A liver chemistry revealed the following (Table 1, Additional file 1): aspartate aminotransferase to alanine aminotranferase (AST/ALT) of 17/17 IU/l, alkaline phosphatase (ALP) of 771 IU/l, lactate dehydrogenase (LDH) of 525 IU/l, and T-Bil and D-Bil markers of 2.63/0.76 mg/dl. Hepatitis B and C virus markers were negative. On palpation, her abdomen revealed a markedly enlarged spleen. A contrast-enhanced computed tomography (CT) scan showed mild hepatomegaly and a markedly severe splenomegaly with dilatation of the splenic vein. An emergency endoscopy showed esophagogastric varices (Li F2 Cb RC (+), Lg-cf F1 RC (-)) (Figure 1). A white thrombus was detected on the middle of the esophageal varices.

**Figure 2:**
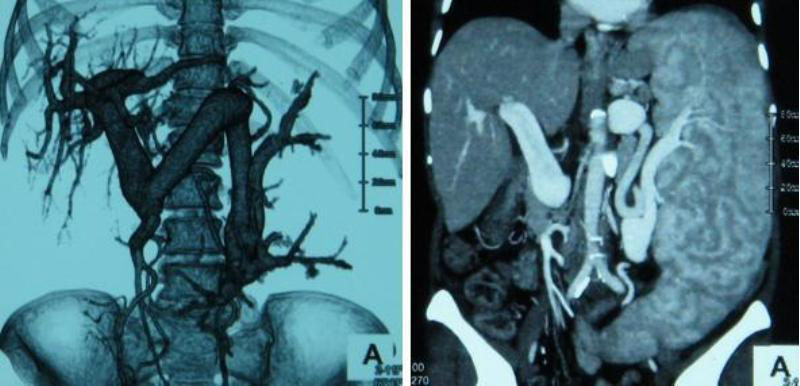
**Abdominal computed tomography**. Dilated splenic vein with massive splenomegaly was demonstrated by a computed tomography scan.

**Figure 3:**
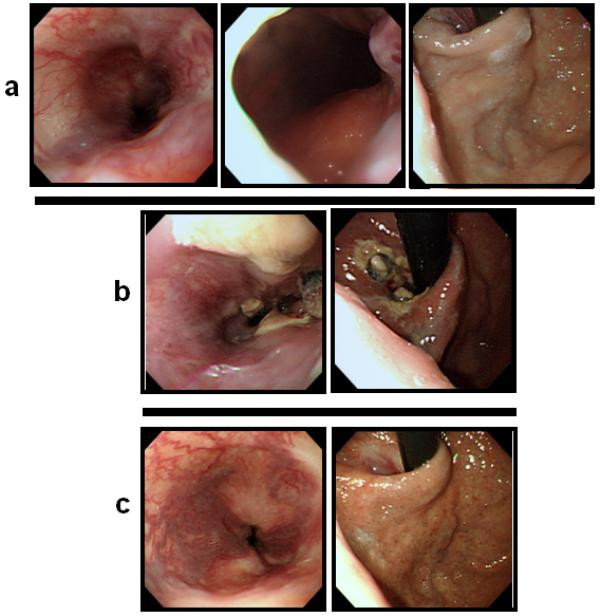
**Endoscopic findings before and after additional endoscopic therapy for the remaining varices**. **(a) **Endoscopic picture shows the remaining esophagogastric varices 41 days after the patient's admission (Ls F3 Cb RC (+), Lg-c F1 Cw RC (-); a variceal ligation with seven rubber bands was carried out. **(b) **Resolving esophageal varices (Lm F1 Cb RC (-), Lg (-) were observed 1 week after the ligation. **(c) **Markedly improved endoscopic findings 4 months after the variceal ligation treatment. The varices were completely eradicated.

**Figure 4:**
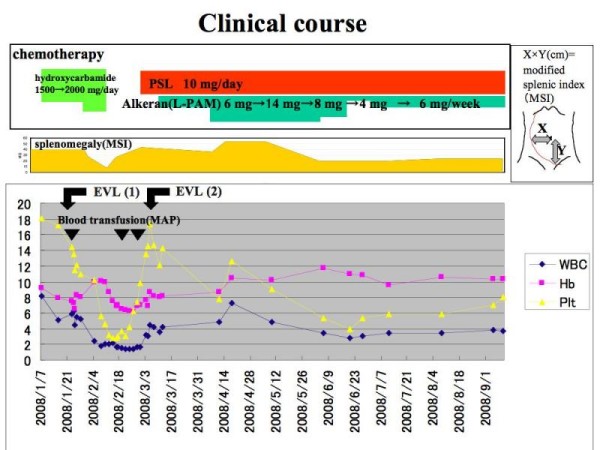
**Clinical course**. Extramedullary hematopoiesis has been treated by chemotherapy using hydroxycarbamide, prednisolone (PSL) and melphalan (Alkelan, L-PAM). After the chemotherapy, the splenomegaly improved without the recurrence of esophagogastric varices. Vertical axis expresses; WBC (/μl), Hb (g/dl), Plt (×10/μl).

The first endoscopic variceal ligation (EVL) with one rubber band was carried out successfully on the first day of her admission. Another endoscopic ligation was performed for the remaining varices 41 days after her admission (Figure 2). In order to eradicate the cause of her varices, the extramedullary hematopoiesis was treated by chemotherapy (hydroxycarbamide, prednisolone and melphalan). After the chemotherapy, her splenomegaly improved without the recurrence of esophagogastric varices (Figure 3 and Figure 4).

## Discussion

In the fibrotic stage of IMF, bone marrow fibrosis results in an ineffective erythropoiesis, thus leading to extramedullary hematopoiesis mainly in the spleen and the liver. We attributed our patient's elevated hepatic venous pressure gradient to the significantly increased wedge hepatic venous pressure, which reflected her profoundly increased intrahepatic sinusoidal resistance. According to results of the splanchnic hemodynamics, which was based on radiological and histological findings, we proposed that obstructed sinusoidal spaces secondary to multiple extramedullary hematopoiesis in the spleen and the liver had a partial role in the development of portal hypertension in our patient. Although an increased splenic blood flow may not be the only mechanism for the development of portal hypertension in our patient, an extremely enlarged spleen may be the main site of red blood cell production. Therefore, an operation with a high risk or radiation for the purpose of a splenectomy might be a poor choice. Although we could have also considered a transjugular intrahepatic porto-systemic shunt (TIPS) [7,8], we chose to perform endoscopic therapy rather than TIPS because of our patient's acute variceal bleeding.

Portal hypertension in patients with IMF may have various causes, such as thrombosis of the hepatic vein, thrombosis of the portal vein, or infiltration of myeloid cells in the liver or spleen [5,9,10]. Any one of these mechanisms might cause intrahepatic portal hypertension followed by variceal bleeding as the complication. Compared to endoscopic injection variceal sclerotherapy, EVL has been reported to have an equal efficacy with lower therapeutic invasion and complications for the management of esophageal variceal hemorrhages [11].

EVL is increasingly used because of its safety and simplicity, and without the necessity of using a sclerosant. A complete eradication of the esophageal varices in our patient was achieved after EVL was performed. However, this therapy is not thought to be a curative treatment for this case because the cause of esophageal varices could be portal hypertension as a complication of extramedullary hematopoiesis in IMF. Therefore, we combined chemotherapy, as shown in Figure 4.

As a result, the size of the modified spleen index decreased and the extramedullary hematopoiesis was suppressed after we increased our patient's dose of hydroxycarbamide to 2000 mg/day. However, the number of blood cells in our patient increased after it had been discontinued. Therefore, prednisolone and melphalan were added to her medication. The extramedullary hematopoiesis was well controlled by this regimen, and her splenomegaly also improved. There has been neither a recurrence of esophageal varices nor deterioration in our patient's hematological problem.

In our patient, the portal hypertension was caused by extramedullary (mainly splenic) hematopoiesis. Since variceal hemorrhaging is an infrequent fatal complication of myelofibrosis, EVL could be considered as therapy of variceal hemorrhages. Moreover, controlling IMF by chemotherapy is an essential treatment for the management of portal hypertension.

## Conclusion

To the best of our knowledge, this is the first case report discussing the successful application of a combination of EVL and chemotherapy as the therapeutic procedure for an esophageal variceal hemorrhage in a patient with myelofibrosis.

## Consent

Written informed consent was obtained from the patient for publication of this case report and any accompanying images. A copy of the written consent is available for review by the Editor-in-Chief of this journal.

## Competing interests

The authors declare that they have no competing interests.

## Authors' contributions

KT, MO, NS and KM analyzed and interpreted the patient data regarding the gastroenterological disease and the endoscopic therapy. MO, SY and SW performed the endoscopic examination of the patient's esophageal varices. MO was a major contributor in writing the manuscript. All authors read and approved the final manuscript.

## Supplementary Material

Additional file 1 **Table 1**. Additional table. Click here for file
