# Supplementary figures and images for: Acute variceal bleeding in a patient with idiopathic myelofibrosis successfully treated with endoscopic variceal band ligation and chemotherapy: a case report
Source: J Med Case Rep. 2010 Jan 28;4:25. doi: 10.1186/1752-1947-4-25 (PMC2830976; doi:10.1186/1752-1947-4-25)

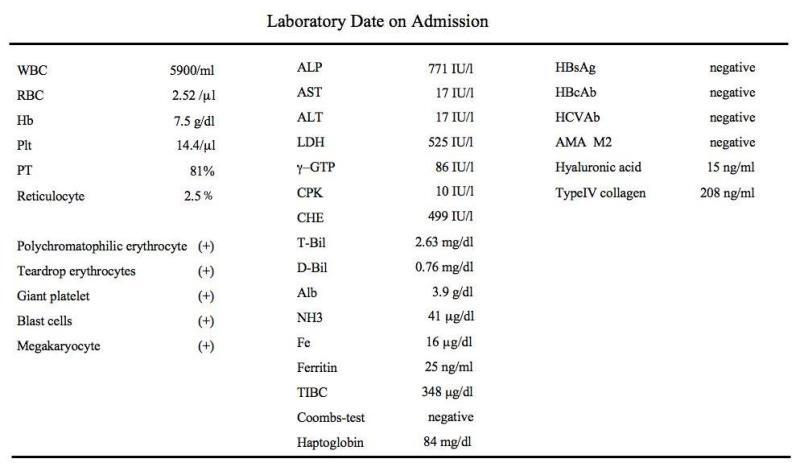

Supplement: Additional file 1 — Table 1. Additional table. [file 1752-1947-4-25-S1.JPEG]
